# Type 2 Diabetes Mellitus Is Associated with More Serious Small Intestinal Mucosal Injuries

**DOI:** 10.1371/journal.pone.0162354

**Published:** 2016-09-06

**Authors:** Hao-Jie Zhong, Yu Yuan, Wen-Rui Xie, Mei-Hui Chen, Xing-Xiang He

**Affiliations:** Department of Gastroenterology, The First Affiliated Hospital of Guangdong Pharmaceutical University, Guangzhou, Guangdong, China; Children's Hospital Boston, UNITED STATES

## Abstract

**Background:**

Clinical and experimental research has revealed that diabetes mellitus (DM) is characterized by intestinal hypomotility, gut microbial dysbiosis, increased gut permeability, microcirculation disorders, circulatory changes, and dysfunction of intestinal stem cells, which may be linked to inflammation of intestinal mucosa. However, the relationship between type 2 DM (T2DM) and macroscopic small intestinal mucosal injuries is still unclear. Therefore, we retrospectively studied capsule endoscopy data to determine the relationship between T2DM and small intestinal mucosal injuries.

**Materials and Methods:**

We compared the records of 38 T2DM patients with those of 152 non-DM patients for small intestinal mucosal injuries. Different types of mucosal injuries and Lewis scores were compared between T2DM and non-DM patients. The relationships between patients with or without different types of diabetic complications and the Lewis score was assessed. Moreover, the relationships between insulin resistance and Lewis score, between HbA1c and Lewis score, were also both assessed.

**Results:**

The prevalence of a villous edema in subjects with T2DM was significantly higher than in those without DM (P < 0.001), but incidence of ulcers was not different (P = 1.000). With T2DM, the Lewis score was also significantly higher (P = 0.002). In addition, subjects with diabetic nephropathy showed significantly higher Lewis scores than patients without diabetic nephropathy (P = 0.033). In Pearson’s correlation tests, the homeostasis model assessment of insulin resistance (HOMA-IR) value was correlated positively with the Lewis score (γ = 0.175, P = 0.015), but no statistical correlation was found between HbA1c level and Lewis score (γ = 0.039, P = 0.697).

**Conclusions:**

Subjects with T2DM, especially those with diabetic nephropathy, have higher Lewis scores and more serious small intestinal mucosal lesions.

## Introduction

Diabetes mellitus (DM) is one of the most common chronic diseases, and its prevalence in China ranges from 8.3 to 12.7% for different regions, as reported in a recent study [1]. DM has been linked to a higher incidence of gastrointestinal symptoms, including stomach ache, diarrhea, and constipation, which is known as diabetic enteropathy [2, 3]. Previous studies have showed that DM is associated not only with carcinomas of the digestive system [4, 5] but also gastrointestinal mucosal injuries such as gastric ulcers [6, 7]. Moreover, clinical and experimental research has revealed that DM is characterized by intestinal hypomotility [8]; gut microbial dysbiosis [9, 10]; increased gut permeability [11, 12]; microcirculation disorders [13]; disorders of circulation; and dysfunction of intestinal stem cells [3], all of which may be linked to inflammation of intestinal mucosa [9].

Injuries of the small intestine have traditionally been neglected, because the incidence of small intestinal diseases is lower than for the stomach and colon. Diagnosis of small intestinal diseases is also more difficult. However, small intestinal injuries can lead to erosion, ulceration, bleeding, and even perforation, which can have serious consequences. Some risk factors for small intestinal mucosal injuries, such as chronic intake of non-steroidal anti-inflammatory drugs (NSAIDs) and cirrhosis of the liver with portal hypertension, have been widely reported [14, 15]. Because adverse events are associated with small intestinal injuries, the identification of risk factors for developing such injuries is of great clinical significance. However, the relationship between type 2 DM (T2DM) and macroscopic small intestinal mucosal injuries is still unclear.

Capsule endoscopy (CE) is widely used to detect small intestinal diseases, due to its ability to provide high-definition images of the small intestinal mucosa and its low miss rate for small intestinal diseases [16]. Therefore, we performed a chart review and utilized CE data to investigate the relationship between T2DM and small intestinal mucosal injuries.

## Materials and Methods

### Ethics statement

The study was reviewed and approved by the First Affiliated Hospital of Guangdong Pharmaceutical University Institutional Review Board. Given that this was a retrospective study, informed consent from the research subjects was waived.

### Patient Selection

We retrospectively reviewed the medical data of 548 consecutive inpatients who underwent CE due to occult gastrointestinal bleeding, abdominal pain, and diarrhea from August 2011 to July 2015 at the First Affiliated Hospital of Guangdong Pharmaceutical University. Of the 548 total patients, 152 were excluded based on the following exclusion criteria: previous diagnosis of carcinoma; recent use of NSAIDs; Crohn’s disease; gastrointestinal infection such as intestinal tuberculosis and acute gastroenteritis; suspected small bowel obstruction; serious heart, lung, kidney (except diabetic nephropathy) or liver disease; and incomplete medication data.

### Methods

Patients fasted for 12 hours and used laxatives for bowel preparation before swallowing the capsule for CE. CE was performed with a PillCam SB1/SB2 capsule (Given Imaging Ltd., Israel). RAPID ACCESS 7.0 software (Given Imaging Ltd.) was used to analyze the CE images.

**Fig 1:**
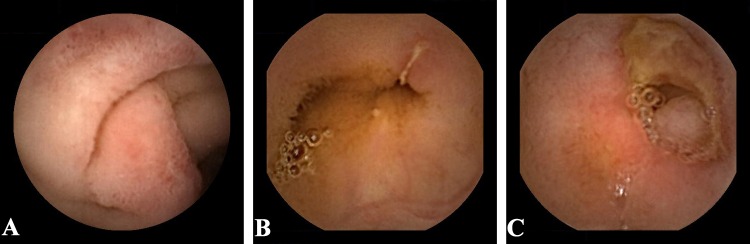
Macroscopic small intestinal mucosal injuries under capsule endoscopy. (A) Villous appearance, defined as edema in which villous width is equal to or greater than villous height. (B) Ulcer, defined as mucosal breaks with white or yellow bases surrounded by red or pink collars. (C) Stenosis.

Small intestinal mucosal injuries were defined as inflammatory change and assessed by the Lewis score, which was created based on three capsule endoscopic variables: villous appearance (edema), ulcers, and stenosis (Fig 1) [17]. The Lewis score is widely used to assessed the small intestinal mucosal injuries, such as Crohn’s disease and inflammatory changes in aspirin users [18]. Based on the algorithm of the score, the small intestine was divided into three tertiles, according to the small intestinal transit time. The score of each tertile was measured by the type, number, longitudinal extent, and descriptors of the mucosal injuries. Similarly, the stenosis score was measured by the number, longitudinal extent, and descriptors of the stenosis. Finally, the maximum tertile score plus the stenosis score was considered to be the total score.

Preliminary analysis indicated that there was an imbalance of age between the T2DM and non-DM subjects. Therefore, age-matched controls were selected from non-diabetic and T2DM subjects, at a 4:1 ratio. Subject patient demographics and clinical characteristics, including gender, age, body mass index (BMI), DM status, DM duration, DM complications (such as cardiovascular or cerebrovascular events, diabetic nephropathy and diabetic peripheral neuropathy); use of anti-diabetic drugs; hypertension status; use of anti-hypertensive drugs; *Helicobacter pylori* (Hp) status; alcohol use and smoking status; and total cholesterol, triacylglycerol, fasting plasma glucose (FPG), fasting insulin (FINS), and hemoglobin A1c (HbA1c) levels were recorded and analyzed. Any information that could identify individual participants (“personal identifiers”) was not recorded.

Based on “stages in diabetic nephropathy” [19], stages of this disease more severe than stage 2 are defined as diabetic nephropathy. In China, criteria for diagnosis of diabetes are as follows: FPG ≥ 7.0mmol/L; or 2-h plasma glucose ≥ 11.1mmol/L during an oral glucose tolerance test; or a random plasma glucose ≥ 11.1mmol/L. Results should be confirmed by repeat testing. The American Diabetes Association’s criterion [20], HbA1c ≥ 6.5%, is not used for diagnosis of diabetes in China. In China, the definition of hypertension is consistent with international criteria [21]. Alcoholism is defined as the ingestion of > 40 g/day of alcohol for 5 years or longer in men, and > 20 g/day in women. And patients who had ever smoked were classified as tobacco smokers. Homeostasis model assessment of insulin resistance (HOMA-IR) values were calculated using the following formula [22]: HOMA—IR = FPG × FINS / 22.5. These medical data were extracted from the subjects’ electronic medical records.

### Statistical analysis

Analyses were performed using IBM SPSS statistics software, version 19.0 (Chicago, IL, USA). Quantitative results from normally distributed data are presented as the mean ± standard deviation. Nonparametric data are presented as medians and interquartile ranges. Categorical data are presented as percentage frequency distributions. The unpaired t-test was used to assess the difference between mean values of the two groups; the Mann-Whitney U-test to assess the difference between medians values of the two groups; and the Chi-square test or the Fisher exact probability for quantitative variables. The Pearson correlation test was used to assess the relationships between HOMA-IR value and Lewis score, and between HbA1c level and Lewis score. A two-tailed P value of less than 0.05 was considered statistically significant.

## Results

### Demographics and clinical characteristics of patients

Based on the medical data, 38 subjects with T2DM and 152 without DM were enrolled. Demographics and clinical characteristics of the subjects are shown in Table 1. The mean duration of DM for the T2DM subjects was 8.47 ± 8.03 years.

**Table 1:** Demographics and clinical characteristics of patients.

Variables	Total	T2DM patients	Non-DM patients	P value
	(n = 190)	(n = 38)	(n = 152)	
**Age (years)**	69.5 (62–76.25)	72 (61–79.25)	69 (62–76)	0.390
**Male gender**	111 (58.4)	21 (55.3)	90 (59.2)	0.659
**BMI (kg/m**^**2**^**)**	22.38 (20.42–24.84)	22.21 (20.68–25.86)	22.43 (20.32–24.61)	0.359
**DM duration (years)**		8.47 ± 8.03		
**Hypertension status**	107 (56.3)	31 (81.6)	76 (50.0)	**<0.001**
**Use of anti-hypertensive drugs**	81 (75.7)	21 (67.7)	60 (78.9)	0.390
	(n = 107)	(n = 31)	(n = 76)	
**Hp infection**	107 (56.3)	22 (57.9)	85 (55.9)	0.826
**Alcoholism**	23 (12.1)	8 (21.1)	15 (9.9)	0.091
**Smoking**	34 (17.9)	5 (13.2)	29 (19.1)	0.394
**TC (mmol/L)**	4.67 ± 1.13	4.65 ± 1.16	4.68 ± 1.13	0.903
	(n = 152)	(n = 34)	(n = 118)	
**TG (mmol/L)**	1.35 (0.86–1.88)	1.55 (0.93–2.12)	1.28 (0.80–1.84)	0.187
	(n = 152)	(n = 34)	(n = 118)	
**HOMA-IR value**	1.72 (0.91–2.52)	2.16 (1.16–4.08)	1.49 (0.89–2.27)	0.062
	(n = 90)	(n = 19)	(n = 71)	
**HbA1c (%)**	5.85 (5.60–6.48)	6.65 (6.10–8.15)	5.70 (5.50–5.98)	**<0.001**
	(n = 60)	(n = 24)	(n = 36)	
**Villous edema**	94 (49.5)	30 (78.9)	64 (42.1)	**<0.001**
**Ulcer**	15 (7.9)	3 (7.9)	12 (7.9)	1.000
**Lewis score**	8.00(0–112)	112 (8–112)	0 (0–112)	**0.002**

Note: Values are presented as mean ± standard deviation, medians (interquartile ranges) or n (%). Abbreviations: BMI: body mass index; DM: diabetes mellitus; Hp: *Helicobacter pylori*; T2DM: type 2 diabetes mellitus; TC: total cholesterol; TG: triacylglycerol; HOMA-IR: homeostasis model assessment of insulin resistance. P values based on comparisons between T2DM and non-DM patients.

### Assessment of type of mucosal injury by CE

The prevalence of a villous edema was 78.9% in T2DM patients, which was significantly higher than that in non-DM patients (P < 0.001). However, the incidence of ulcers showed no statistical difference between the two groups (P = 1.000). No obvious stenosis was found in any of the enrolled patients.

### T2DM and Lewis score

In patients with T2DM, the median (interquartile ranges) of Lewis score was 112 (8–112), which was significantly higher than for non-DM patients (P = 0.002).

Because of the imbalance of hypertension status between T2DM and non-DM patients, subgroup analysis was used to modify the effect of hypertension status and to determine if T2DM affects Lewis score. In the subgroup of patients with hypertension, the median Lewis score in T2DM patients was significantly higher than for non-DM patients at 112 (8–112) than at 0 (0–112), P = 0.038. Similarly, in the subgroup of patients without hypertension, the median Lewis score in T2DM patients was also significantly higher at 112 (8–247) vs. 0 (0–112) P = 0.047.

### Diabetic complications and Lewis score

The relationships between patients with or without different types of diabetic complications and the Lewis score are presented in Table 2. Patients with diabetic nephropathy showed significantly higher Lewis scores than patients without diabetic nephropathy (P = 0.033). In contrast, a history of cardiovascular or cerebrovascular complications, or diabetic peripheral neuropathy, did not appear to influence Lewis scores.

**Table 2:** Lewis score in diabetic patients with or without different types of complications.

	Lewis score	P value
**Cardiovascular complication**		0.401
**Yes (n = 19)**	112 (8–112)	
**No (n = 19)**	112 (8–112)	
**Cerebrovascular complication**		0.296
**Yes (n = 6)**	112 (86–145.75)	
**No (n = 32)**	112 (2–112)	
**Diabetic peripheral neuropathy**		0.337
**Yes (n = 6)**	56 (0–118)	
**No (n = 32)**	112 (8–112)	
**Diabetic nephropathy**		**0.033**
**Yes (n = 3)**	136 (112-absent)	
**No (n = 35)**	112 (8–112)	

Note: P values based on comparisons between T2DM patients with or without complications.

### Anti-diabetic drugs and Lewis score

In 38 T2DM patients, 6 patients used no anti-diabetic drugs and 32 patients used anti-diabetic drugs (24 patients took oral anti-diabetic drugs, 4 patients used insulin, and 4 patients used both oral agents and insulin).

However, the Lewis score showed no statistically significant difference between patients who used anti-diabetic drugs and those who did not (P = 0.831). In addition, the Lewis score also showed no statistically significant difference among patients with the use of oral anti-diabetic drugs, insulin, and both oral drugs and insulin (P = 0.579).

### Relationship with HOMA-IR values and HbA1c levels

We were able to retrieve medical data for the HbA1c levels of 104 patients, and calculated HOMA-IR values for 193 patients. The relationships between HOMA-IR value and Lewis score and between HbA1c level and Lewis score were analyzed and the results are listed in Table 3. In the Pearson correlation test, the HOMA-IR value was correlated positively with the Lewis score (γ = 0.175, P = 0.015). However, there was no statistical correlation between HbA1c level and Lewis score (γ = 0.039, P = 0.697).

**Table 3:** Correlation test for Lewis score.

	γ	γ^2^	P value
**HOMA-IR value**	0.175	0.031	**0.015**
**(n = 193)**			
**HbA1c (%)**	0.039	0.002	0.697
**(n = 104)**			

Abbreviations: HOMA-IR: homeostasis model assessment of insulin resistance.

## Discussion

### T2DM and mucosal injuries

In this retrospective study, we found that the subjects with T2DM had a higher prevalence of villous edema of the small intestine, and higher Lewis scores than the non-diabetic subjects. These findings show that T2DM is associated with more-serious small intestinal mucosal injuries. To the best of our knowledge, this is the first study to demonstrate an association between T2DM and macroscopic small intestinal mucosal injuries.

Among previous clinical and experimental studies, some investigations linked T2DM to small intestinal inflammatory changes that could partly explain the results of the present study. Firstly, small intestinal mucosal injuries could be caused by gut microbial dysbiosis in diabetic patients. In support of this, a metagenome-wide association study that analyzed gut microbial content found that some butyrate-producing bacteria were significantly reduced, and various opportunistic pathogens increased, in patients with T2DM [10]. In addition, Sato *et al*. [23] detected gut bacteria in the circulation at a significantly higher rate in diabetic individuals, indicating that gut bacteria translocate from the intestine to the bloodstream. Moreover, Larsen *et al*. [24] showed that *Betaproteobacteria* and *Bacteroidetes* were enriched, whereas the proportion of *Firmicutes* was markedly decreased in diabetic compared with non-diabetic individuals. Gut endocannabinoid expression (which could affect gut permeability) and tight junction proteins were modulated by intestinal microbiota in T2DM mice [9, 25, 26]. Similarly, increased gut permeability was found in humans with T2DM [27]. Changes in gut permeability and tight junction proteins caused bacterial translocation and endotoxemia, which could induce low-grade inflammation in the intestines [28]. Secondly, one of the complications of diabetes, microangiopathy, might account for inflammatory changes in the small intestine. Indeed, Sambuceti *et al*. [29] reported that arterial recruitment of endothelial progenitor cells was reduced in diabetic mice, which might be caused by a reduction of serum adiponectin and phosphorylated AMP-activated protein kinase. In addition, elevated free fatty acid levels in plasma of T2DM patients could lead to the release of proinflammatory cytokines and the generation of lipid metabolites, all of which are risk factors for vascular inflammation and microangiopathy [30]. Subsequent disruption of the microcirculation may then induce injuries to small intestinal mucosa. Thirdly, diabetic neuropathy could also cause intestinal injuries. Diabetic patients showed a higher prevalence of impaired intestinal motility, which was attributed to autonomic neuropathy [31]. Intestinal motility disorders are often followed by gut microbial dysbiosis and bacterial translocation, which may also lead to mucosal damage [32]. Fourthly, the function of intestinal stem cells might be disrupted in DM patients. A recent study showed that the levels of insulin-like growth factor 1 (IGF-1) and insulin-like growth-factor-binding-protein (IGFBP-3) are altered in DM patients [3]. Altered levels of these circulating factors disrupted the function of colonic stem cells (CoSC), which is linked to reductions of intestinal crypt numbers and decreases in epithelial-cell proliferation [33]. Thus, resolution of mucosal inflammation and injuries in the colon was difficult, and similar phenomena might also occur in the small intestinal mucosa.

Despite the association between T2DM and higher Lewis scores, the prevalence of ulcers showed no significant differences between T2DM and non-diabetic patients (which means that the small intestinal mucosal inflammation did not result in very serious injury in the current study). There are three possible explanations for these results. Firstly, when the microcirculation of the small intestine is impaired, the richness of arterial communication branches and blood supply plays a compensatory role to prevent serious injuries. Secondly, diabetic complications, including microangiopathy and neuropathy, often occur after more than 10 years following diagnosis of diabetes [34, 35]; however, the mean duration of diabetes in the diabetic patients of the current study was only 8.47 years, so serious intestinal injuries followed by vascular and neural complications may not yet have occurred. Thirdly, the small sample size and potential confounders, such as intestinal preparation quality, might have affected the results.

### Diabetic complications and mucosal injuries

The results of this study revealed that T2DM patients with diabetic nephropathy seem to suffer more serious intestinal mucosal injuries compared with those without diabetic nephropathy. Several investigations suggested that there was impaired small intestinal motility [36], impaired intestinal barrier function [37], lower number of crypts, and reduction in epithelial cell in patients with chronic renal insufficiency [3]. In addition, patients with diabetic nephropathy might have more-serious diabetic neuropathy and microangiopathy, which are linked to serious mucosal injuries. Thus, diabetic nephropathy patients with symptoms of diabetic enteropathy might be a new indicator for undergoing CE examination. However, the severity of mucosal injuries showed no obvious relationship to cardiovascular events, cerebrovascular complications, or diabetic peripheral neuropathy, in T2DM patients

### Anti-diabetic drugs and mucosal injuries

The results revealed no statistical relationship between the use of anti-diabetic drugs and mucosal injuries in T2DM patients. However, the small sample size might not be large enough to detect a statistical difference. In addition, the duration of anti-diabetic drug use in DM patients was unclear, which might also influence the result. Thus, a large-scale and rigorous study will be needed to investigate the relationship between the use of anti-diabetic drugs and mucosal injuries.

### Relationship with HOMA-IR values and HbA1c levels

No statistical correlation was found between HbA1c level and Lewis score, which may also be partly due to the small sample size. More importantly, small intestinal mucosal injuries that were linked to the diabetic vascular or neurological complication were based on not only the status of controlling blood glucose, but also a long duration of disease. However, HbA1c only represented the average blood glucose level over the prior 2–3 months.

Interestingly, we found that the insulin HOMA-IR value was positively correlated with Lewis score. Moreno-Navarrete *et al*. [38] demonstrated that circulating zonulin, a physiological mediator that regulates intestinal permeability, was increased in patients with insulin resistance[38]. As described above, increased intestinal permeability could induce intestinal inflammation through the translocation of the endotoxin and bacteria, which may partly explain the relationship between insulin HOMA-IR values and Lewis score. As has been well established, insulin resistance is associated not only with diabetes, but also with obesity, fatty liver disease, and metabolic syndrome. Based on these considerations, we sought to clarify whether these disorders have similar outcomes with regard to intestinal injury.

### Limitations

There are several limitations to the present study. Firstly, there may have been selection biases, because we only included inpatients (who may correspond to patients with a more serious state of illness). Secondly, this was a single-center study with a small sample size, and the duration of diabetes might not have been sufficient to reveal differences in the incidence of ulcers between T2DM and non-DM patients. Thirdly, potential confounders such as fatty liver disease, metabolic syndrome, and use of other drugs were not taken into consideration; thus, the small intestinal injuries might partly have been influenced by these confounders. Fourthly, as the study was retrospective, some confirmatory analyses, such as assessment of symptoms with questionnaire and pathology analysis on bioptic samples, were hard to achieve. Our conclusions on the effect of T2DM on small intestinal mucosal injuries should therefore be interpreted with caution, and large-scale, multi-center, and perspective studies will be needed to confirm these conclusions.

## Conclusion

This study suggests that T2DM is associated with significant small intestinal mucosal injuries, based on a higher Lewis score. Our findings also suggest that if occult gastrointestinal bleeding, abdominal pain, and diarrhea are found in T2DM, especially diabetic nephropathy patients who have been examined by gastroscopy and colonoscopy, it is imperative to ascertain by the use of CE whether these adverse events are a result of small intestinal mucosal injuries. Moreover, weight loss can alleviate insulin resistance, and may also play an essential role for treatment of small intestinal mucosal injuries in patients with T2DM.
